# *Phlebotomus duboscqi* gut microbiota dynamics in the context of *Leishmania* infection

**DOI:** 10.3389/fimmu.2025.1717935

**Published:** 2026-01-05

**Authors:** Kristina Tang, Yue Zhang, Claudio Meneses, Luana A. Rogerio, Laura Willen, Eva Iniguez, Shaden Kamhawi, Jesus G. Valenzuela, Fabiano Oliveira, Pedro Cecilio

**Affiliations:** 1 Vector Molecular Biology Section, Laboratory of Malaria and Vector Research, National Institute of Allergy and Infectious Diseases, National Institutes of Health, Rockville, MD, United States; 2 Integrated Data Sciences Section (IDSS), National Institute of Allergy and Infectious Diseases, National Institutes of Health, Rockville, MD, United States; 3 Vector Biology Section, Laboratory of Malaria and Vector Research, National Institute of Allergy and Infectious Diseases, National Institutes of Health, Rockville, MD, United States

**Keywords:** sand fly, *Leishmania* infection, gut microbiota, relative abundance, diversity

## Abstract

**Introduction:**

The manipulation of the gut microbiota of disease vectors has emerged as a new approach to use in the integrated control of vector-borne diseases. For this purpose, a deep knowledge of their gut microbial communities is essential. To our knowledge, to date, no study has documented the gut microbiome dynamics of *Phlebotomus duboscqi* sand flies over the entire time-period required for the maturation of a *Leishmania* infection. Here, we address this limitation.

**Methods:**

*P. duboscqi* midguts were dissected both before and at different days after *L. major* infection and subjected to genomic DNA extraction followed by amplification of the V3-V4 hypervariable regions of the 16S rRNA, sequencing, and metagenomics analysis.

**Results:**

We observed a decrease in the number of Amplicon Sequence Variants (ASVs) early after infection, at D2, and late after infection, at D12. More so *Sphingomonas*, *Ochrobactrum*, and *Serratia* emerged as the most prevalent genera in relative terms, before, early after, and late after infection, respectively. These results translated into a separation between the 3 groups in the context of a beta diversity analysis, with statistical relevance. Importantly, we were able to establish *Corynebacterium* spp. and *Enterococcus* spp. as potential markers of non-infected and infected sand flies, respectively, as well as *Streptococcus* spp., *Sphingomonas* spp., *Ralstonia* spp., and *Abiotrophia* spp. as potential specific markers of late infections (ANCOM-BC analysis).

**Discussion:**

Overall, we show that the composition of the gut microbiota of *P. duboscqi* sand flies changes significantly over the course of an infection with *L. major* parasites.

## 1 Introduction

Although often not recognized as so, sand flies are among the insects of significant public-health concern; they show a worldwide distribution, depend on blood to complete their life cycle, and can transmit different pathogens (1). Among these, *Leishmania* parasites, the causative agents of human leishmaniasis, are the ones associated with the highest disease/socioeconomic burden (2, 3); over 1.000.000 leishmaniasis cases are estimated to occur yearly, most referring to the non-fatal cutaneous form (4). Of note, most human-infecting *Leishmania* spp. are zoonotic agents (1), a vaccine for human leishmaniasis is yet not available (5), and the anti-*Leishmania* chemotherapeutics are limited and increasingly compromised by the emergence of drug resistance (6). Therefore, the control of leishmaniasis is extremely challenging, requiring a multifaceted approach that needs to have a major focus on the sand fly vector, instead of relying solely on case-control strategies.

While there are a few sand fly-based approaches in use for the control of leishmaniasis, they all aim to prevent sand fly-human contact (e.g. repellents/insecticides) (7). However, these have limitations, including the emergence of resistance (7). Of note, contrary to the reality for other disease vectors (8), no control method targeting specifically the infection within sand flies is available for application (7). Yet, recent studies reported that, if depleted from their natural gut microbiota, sand flies are unable to sustain the development of *Leishmania* spp. parasites within their midguts (9, 10). This means that, likely, there are bacteria that promote the establishment of *Leishmania* in sand flies, and thus, that we should be able to make sand flies refractory to infection via the manipulation of their gut microbiota. Such interventions will either directly target the promoters of *Leishmania* growth, or depend on the introduction of alternative agents that, either directly or indirectly, are detrimental for the development of *Leishmania* parasites in the vector. In fact, our recent work shows that the latest is possible. By introducing a mosquito symbiont, *Delftia tsuruhatensis* TC1, in the sand fly midgut, we induced a state of sand fly gut dysbiosis that negatively impacted the development of *Leishmania major* parasites in the vector, and, consequently, their transmission to hosts (11).

For the development of similar approaches, there is the need for a comprehensive understanding of the composition and dynamics of the gut microbiota of sand flies. However, while the gut microbiota of some species of medical importance, including *Lutzomyia longipalpis* and *Phlebotomus papatasi*, has been extensively characterized, the information available on the subject for other species is limited (12, 13). That is the case of *Phlebotomus duboscqi* sand flies, the main West African vectors of *L. major*-caused cutaneous leishmaniasis (14); only four studies looked at their gut microbiota (colony-reared sand flies) (10, 15–17). The older studies focused either on culturable bacteria (17), or on temperature-gradient gel electrophoresis of whole DNA (16) and only in the context of non-infected sand flies. The most recent studies used more modern 16S-based sequencing methods, including in the context of infected sand flies, but looking only at a single time-point after infection [either early (15) or late (10)]. To our knowledge, no study documented the gut microbiome dynamics of *P. duboscqi* sand flies over the entire time-period required for the maturation of a *Leishmania* infection. Here, to address this limitation, we looked at the differences of the gut microbiome of laboratory-reared *P. duboscqi* sand flies before and after *L. major* infection, with the necessary temporal resolution to understand the dynamics of sand fly gut microbial communities throughout the infection timeline.

## 2 Methods

### 2.1 Ethics statement

All animal experiments were carried out in accordance with the NIAID Animal Care and Use Committee under the animal protocol LMVR4E.

### 2.2 Parasites, sand flies, and infection

A cloned line of *Leishmania major* (WR 2885) was used (18). Promastigotes were maintained at 26°C in Schneider’s insect medium with 20% heat-inactivated FBS and 100 U/mL penicillin/streptomycin (all Thermo Fischer Scientific).

*Phlebotomus duboscqi* sand flies were mass reared at the LMVR insectary as previously (19). After an overnight starving period, sand flies were infected by artificial feeding on defibrinated rabbit blood (Spring Valley Laboratories) containing *L. major* promastigotes (5x10^6^/ml), as previously (20). Blood-fed females were then sorted and kept on 30% sucrose.

### 2.3 Metagenomics analysis – layout and samples

Sand fly midguts were dissected under a sterile-like environment (21) at different time-points: before blood feeding (D0), and different days after blood feeding (D2, D5, D7, D9, and D12). Dissected midguts were washed three times with sterile PBS and pooled into Eppendorf tubes; 20 midguts per condition were collected, at least in triplicate. Genomic DNA was then extracted and the samples were subjected to 16S rRNA amplification and sequencing (~100,000 reads per sample) as previously (9); the V3-V4 hypervariable regions of the 16S rRNA was targeted with the primers 341F-CCTAYGGGRBGCASCAG, and 806R-GGACTACNNGGGTATCTAAT. Altogether, 21 samples were analyzed.

### 2.4 Metagenomics analysis - amplicon sequence variant calling, phylogenetic tree, and taxonomy classification

This paper results from a re-analysis of the data collected in a different study (11), previously deposited at NCBI under the BioProject number PRJNA1079352 (https://www.ncbi.nlm.nih.gov/bioproject/PRJNA1079352/con); only CTRL samples are relevant to the present study. The initial analysis steps were common to the ones previously reported (code available at https://github.com/GaryZhangYue/Cecilio_2024_TC1_sandflies).

Briefly, 16S rRNA amplicon reads were demultiplexed and trimmed using Novogene in-house scripts. These reads were then imported into QIIME2 v.2021.4 (22) for downstream analysis. DADA2 (23) was used to call Amplicon Sequence Variants (ASVs). Chimeric sequences showing considerably lower abundance than their parent sequences were identified and removed by setting the flag “–p-min-fold-parent-over-abundance” to 10. A rooted phylogenetic tree was generated using FastTree (24) based on a multiple alignment with MAFFT (25). The ASVs were taxonomically classified with a Naïve Bayes classifier pre-trained on the SILVA rRNA database (release 138 SSURef NR99) (26). After DADA2 quality filtering, and considering only the CTRL samples, a total of 3,477,690 reads (165,604±4,990 reads/sample) were retained. The samples were then rarefied to a subsampling depth of 149,894 reads/sample to ensure an even sequencing depth. After rarefaction, 1,844 ASVs and all samples were retained.

### 2.5 Metagenomics analysis - microbial diversity calculation, statistical testing, and differential abundance testing

Diversity metrics were calculated using the QIIME2 core-metrics-phylogenetic function. The rarefied features’ table was used to compute the Shannon’s (27), observed features, Faith’s PD (28), and Pielou’s (29) metrics. Statistical comparisons in this context were made using the Kruskal-Wallis test followed by a *post-hoc* analysis, when applicable, using the Dunn’s test. To visualize the dissimilarities in microbial communities across groups, the weighted UniFrac distance metrics (30) was used to generate PCoA coordinates after Cailliez transformation (31) to correct for negative eigenvalues. Permutational multivariate analysis of variance (PERMANOVA) (32) and permutational multivariate analysis of group dispersion homogeneity (PERMDISP) (33) were applied to compare the centroid location and within-group dispersion level, respectively, across groups. Analysis of compositions of Microbiomes with Bias Correction (ANCOM-BC, version 2.0.3) (34) was also used to find differentially abundant genera between conditions on each day and between each pair of phases (early after *versus* before infection, late after *versus* before infection, and late *versus* early after infection). The unrarefied features’ table was used as input to ANCOM-BC as per the standard recommendations.

## 3 Results

To investigate the dynamic changes in *P. duboscqi* gut microbial communities in the context of *Leishmania* infection, pools of sand fly midguts were collected one day before (day 0), as well as 2-to-12 days post-*L. major* infection and subjected to metagenomics analysis. The infection followed a healthy pattern, with the number of parasites per midgut plateauing after day 7 of infection (**Supplementary Figure S1A**), and the frequency of metacyclic parasites increasing dramatically from day 7 onward (**Supplementary Figure S1B**). For analysis purposes, samples were either considered by individual time-point, or joined together in the frame of three major groups: before infection, early after infection (days 2/5 post-infection, when blood/blood remnants may still be found in the midgut), and late after infection (days 7-to-12 post-infection, when metacyclic promastigotes are present in the sand fly midgut; **Supplementary Figure S1B**).

**Figure 1:**
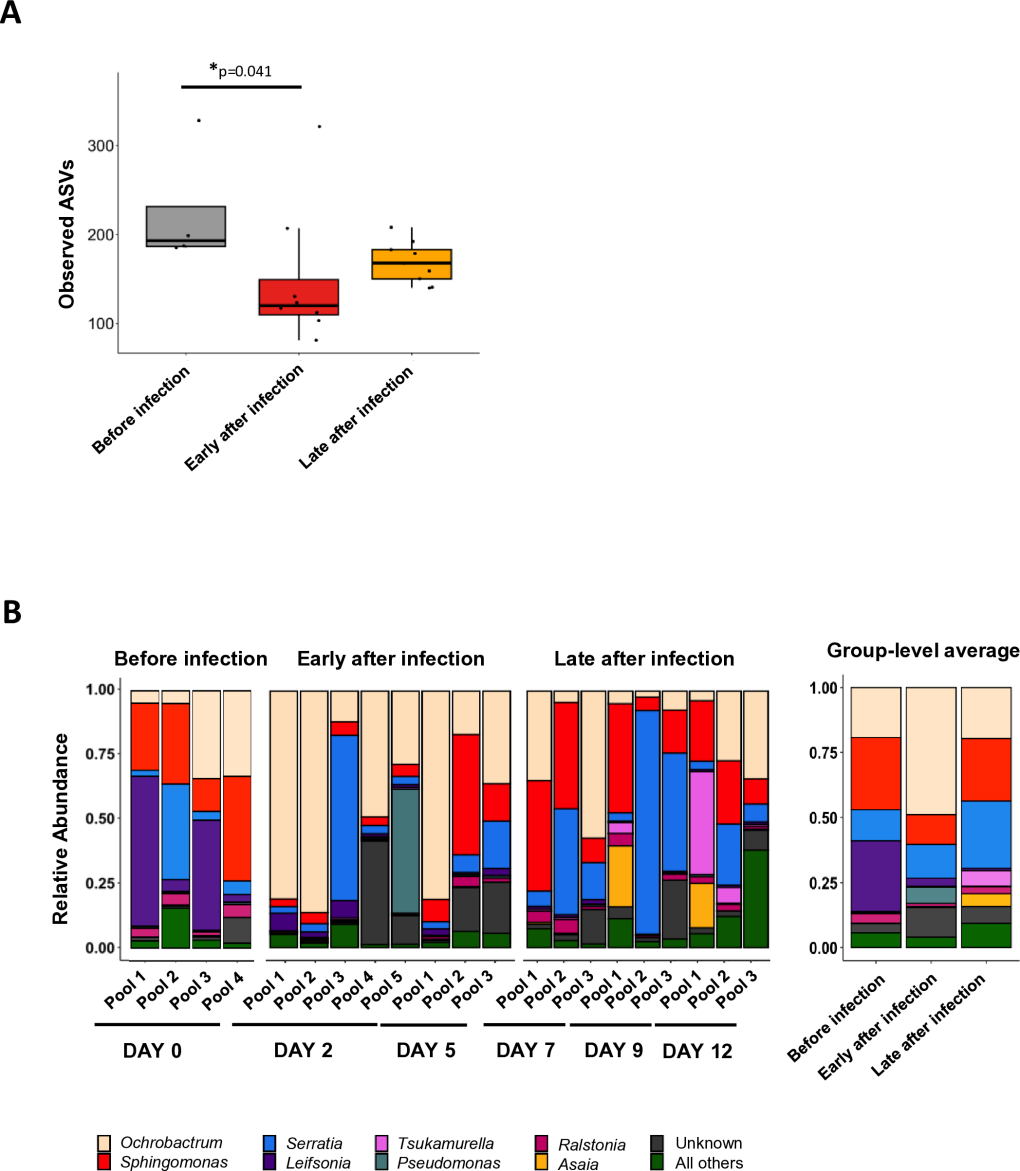
Observed ASVs in function of the infection status and relative abundance at the genus level. Pools of P. *duboscqi* sand fly midguts were collected one day before (day 0), as well as 2, 5, 7, 9, and 12 days after infection with *L. major* parasites and subjected to metagenomics analysis. **(A)** Variation in the number of observed Amplicon Sequence Variants ASVs with the status of infection. Box-and-whisker graphs show an overview of the calculated values per pool of sand fly midguts collected before infection (day 0; grey; n=4), early after *Leishmania* infection (days 2 and 5; red; n=8), and late after *Leishmania* infection (days 7, 9, and 12; orange; n=9). Statistical significance was determined using the Kruskal-Wallis test followed by *post-hoc* analysis and is highlighted. The complete statistical analysis results are listed in **Supplementary Table S1**. **(B)** Relative abundance at the genus level per sample pool and time-point (left panel), as well as per infection status (right panel). The most abundant families are color-coded. All results were obtained in three independent experiments.

First, we looked at the number of observed ASVs as a reflection of the bacterial richness. A significant difference was detected, particularly when we compared the sand fly gut microbiota before and early after infection, with a lower number of ASVs observed in the latter group (**Figure 1A**; **Supplementary Table S1** - adjusted p=0.041). This was mostly due to a decrease in the number of ASVs 2 days post-infection (**Supplementary Figure S2A**; **Supplementary Table S2**). Later after infection, a partial recovery in the number of observed ASVs was evident, although to median group levels still lower than those observed before infection (**Figure 1A**). Considering the individual time-points pertaining to this period, a tendency of reduction of the number of observed ASVs was observed at D12 (**Supplementary Figure S2A**).

Next, we investigated the abundance of the 10 most relevant bacterial groups found in the gut microbiota of *P. duboscqi* sand flies throughout the *Leishmania* infection timeline. Of note, some heterogeneity was visible, as expected, since each sample derives from a pool of midguts. Before infection, more than 85% or relative abundance was attributed to 4 main genera: *Sphingomonas*, *Leifsonia*, *Ochrobactrum*, and *Serratia* (27.7%, 27.2%, 19.3%, 12.0%, respectively; **Figure 1B**, right panel; **Supplementary Table S3**). This translated into the dominance of 4 main families before *Leishmania*-infection (each including one of the abovementioned genera, in the respective order): Sphingomonadaceae, Microbacteriaceae, Rhizobiaceae, and Yersiniaceae (**Supplementary Figure S2B**, right panel; **Supplementary Table S4**).

Early after infection, the most evident change was an increase in the relative abundance of the *Ochrobactrum* genus (and the Rhizobiaceae family) to around 50% (**Figure 1B**; **Supplementary Figure S2B**; right panels). Additionally, two other families, Alcaligenaceae (no genus attributed) and Pseudomonadaceae (referring to the *Pseudomonas* genus) also increased in abundance to values above 6% at the group level; in the latter case, this was mostly due to the contribution of one sample (**Figure 1B**; **Supplementary Figure S2B**; left panels; **Supplementary Tables S3**, **S4**). Conversely, all other abundance-wise relevant genera/families maintained or decreased their prevalence early after infection at the group level. *Leifsonia* (Microbacteriaceae family) contracted the most in relative terms, followed by *Sphingomonas* (Sphingomonadaceae family); for the sake of comparison relative abundance values at the genus level dropped to 3.0%, and 11.3%, respectively as compared to values of 27.2% and 27.7%, before infection (**Figure 1B**; **Supplementary Figure S2B**; right panels; **Supplementary Tables S3**, **S4**). Of note, *Serratia* (Yersiniaceae family) seemed to maintain a relative abundance weight (versus before infection) of around 13% (**Figure 1B**; **Supplementary Figure S2B**; **Supplementary Tables S3**, **S4**).

Late after infection both the *Ochrobactrum* and *Sphingomonas* genera (and their respective families) returned to the relative abundance levels observed before infection, with a contraction of the former and an expansion of the latter (versus early after infection; **Figure 1B**; **Supplementary Figure S2B**). The same was true for the Alcaligenaceae and Pseudomonadaceae families; both lost relevance later after infection, returning to the low relative abundance values (below 1%) detected before infection (**Supplementary Figure S2B**; **Supplementary Table S4**). On the other hand, *Leifsonia* (Microbacteriaceae family) did not recover, maintaining an even lower relative abundance late after infection (e.g. at the genus level, 0.9% versus 3.0% early after infection; **Figure 1B**; **Supplementary Figure S2B**; **Supplementary Tables S3**, **S4**). In contrast, 3 genera/families increased in relative abundance to levels above those detected for both the pre-infection and early infection statuses. *Serratia* (Yersiniaceae family) became the most prevalent late after infection (25.8% both at the genus and family level; **Figure 1B**; **Supplementary Figure S2B**; **Supplementary Tables S3**, **S4**). Additionally, the *Tsukamurella* and *Asaia* genera (Tsukamurellacea and Acetobacteraceae families, respectively) also increased in relative abundance from less than 0.5% before/early after infection to around 5% late after infection (**Figure 1B**; **Supplementary Figure S2B**; right panels; **Supplementary Tables S3**, **S4**). Interestingly, both were detected in higher abundance from day 9 post-infection onward (**Figure 1B**; **Supplementary Figure S2B**; left panels). Of note, one other genus (family), *Ralstonia* (Burkholderiaceae) showed relevant, although lower relative abundance values throughout the experimental timeline; these contracted from 3.6% before infection to 1.1% early after infection and then expanded to 2.8% late after infection (**Figure 1B**; **Supplementary Figure S2B**; **Supplementary Tables S3**, **S4**). The combined relative abundance of all remaining genera (families) varied from around 6% before infection to around 9% late after infection.

**Figure 2:**
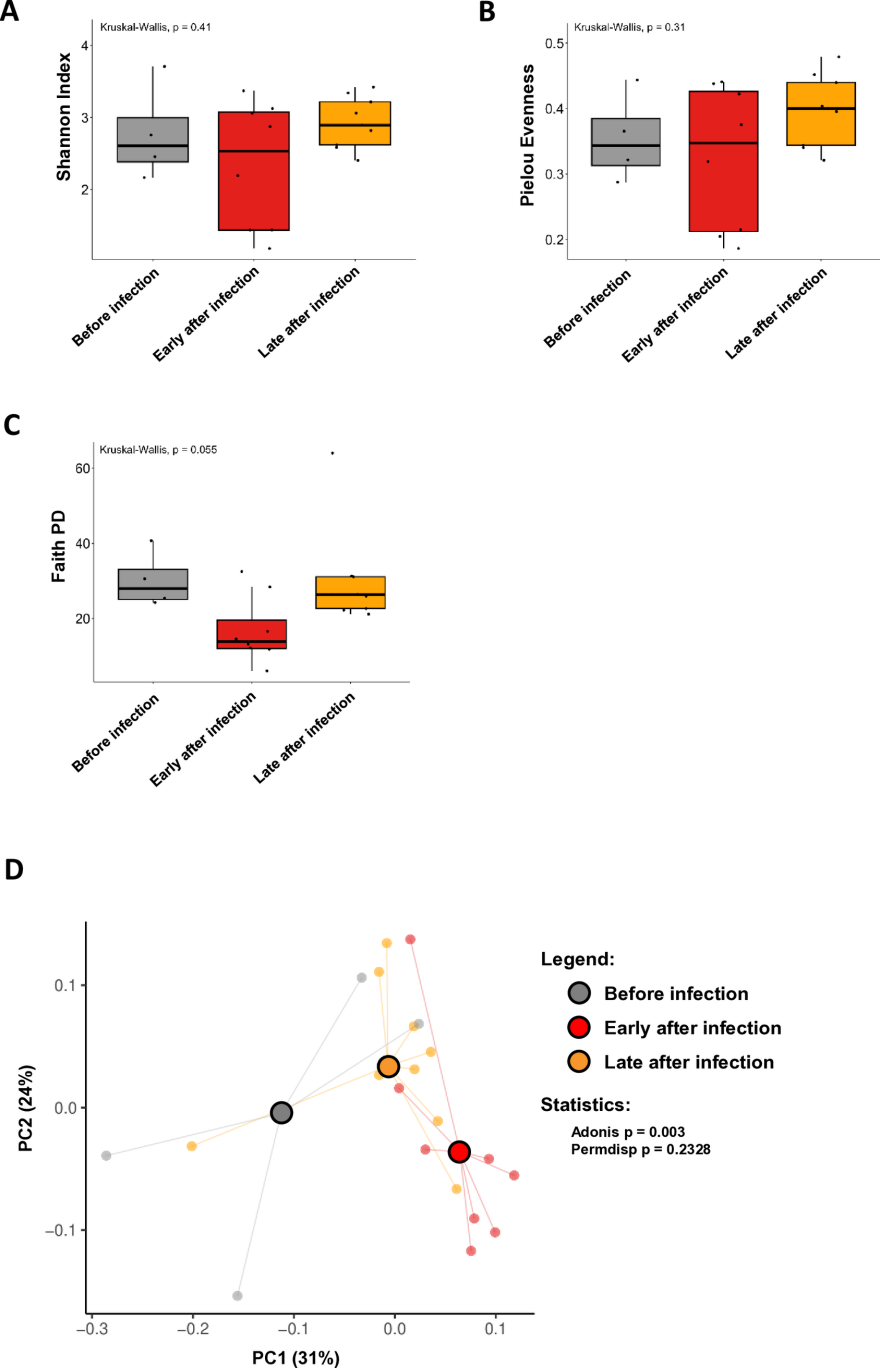
Bacterial diversity metrics. Pools of P. *duboscqi* sand fly midguts were collected one day before (day 0), as well as 2, 5, 7, 9, and 12 days after infection with *L. major* parasites and subjected to metagenomics analysis. Three different indicators were used to evaluate the alpha diversity at the infection status level: **(A)** Shannon Index, **(B)** Pielou Evenness, and **(C)** Faith PD. Box-and-whisker graphs show an overview of the calculated values per pool of sand fly midguts collected before infection (day 0; grey; n=4), early after *Leishmania* infection (days 2 and 5; red; n=8), and late after *Leishmania* infection (days 7, 9, and 12; orange; n=9). Statistical significance was determined using the Kruskal-Wallis test followed by *post-hoc* analysis and is highlighted. **(D)** PCA plot referring to the beta-diversity weighed analysis at the infection status level. Each dot represents and individual sample, which is color coded: samples collected before (day 0), early (days 2 and 5) after, and late (days 7, 9, and 12) after *Leishmania* infection are highlighted in grey, red, and orange, respectively. Statistical significance (permutational ANOVA and beta dispersion analyses) is denoted in the graph. Complete statistical analysis results, including between-group comparisons, are listed in **Supplementary Table S5**. All results were obtained in three independent experiments.

The above-reported changes highlighted possible dynamic alterations in the sand fly midgut microbiota composition, in the context of *Leishmania* infection. Therefore, next we looked specifically at bacterial diversity. With respect to alpha diversity metrics, referring to within-sample diversity (richness/evenness), no statistically significant differences were observed (**Figures 2A–C**). Regarding both Shannon index and Pielou evenness metrics, the distribution of individual samples overlapped group-wise (**Figures 2A, B**). Regarding the Faith PD metric (**Figure 2C**), the profile was similar to that of the Observed ASVs (**Figure 1A**), but without statistical relevance. On the other hand, with respect to beta diversity metrics (weighted UniFrac distance), reflecting the overall composition at the group level, statistically significant differences were detected. A group separation was evident [p(adonis)=0.003], and to a greater extent between before infection and early after infection samples (**Figure 2D**). This translated into significant differences when we compared specifically the before infection and early after infection groups [p(PERMANOVA)=0.024; **Supplementary Table S5**], as well as almost significant differences when we compared the early after infection and late after infection groups [p(PERMANOVA)=0.054; **Supplementary Table S5**]. Of note, no major differences in terms of sample dispersion within groups (or group heterogeneity) were observed [p(betadisp)>0.2 and p(PERMDISP)>0.2; **Figure 2D**; **Supplementary Table S5**]. Overall, these results suggest that the composition of the sandfly gut microbiota changes dynamically with the progression of *Leishmania* infection in sand flies.

**Figure 3:**
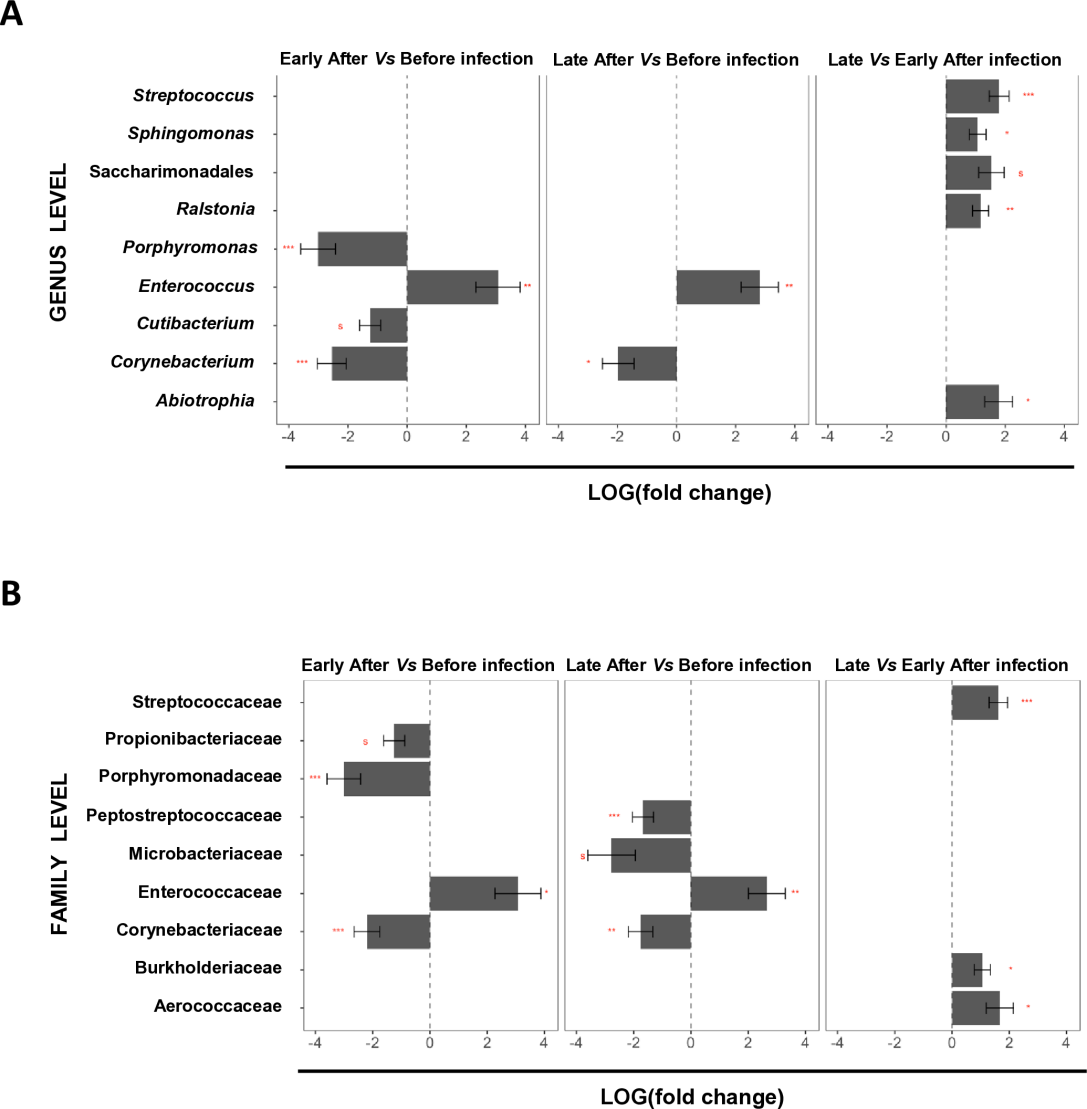
Absolute abundance-based infection stage-specific markers. Pools of P. *duboscqi* sand fly midguts were collected one day before (day 0), as well as 2, 5, 7, 9, and 12 days after infection with *L. major* parasites and subjected to metagenomics analysis. Analysis was done in the frame of three major groups: before infection (day 0), early after infection (days 2 and 5), and late after infection (days 7, 9, and 12). **(A)** Significant changes in the estimated absolute abundance of different bacterial genera in the sand fly midgut early after *versus* before *Leishmania* infection (left panel), late after *versus* before *Leishmania* infection (center panel), and late *versus* early after *Leishmania* infection (right panel). Data are represented by effect size (LogFC) and Standard Error bars (two-sided; Bonferroni adjusted) derived from the ANCOM-BC model. All effect sizes with adjusted p < 0.1 (q value) are indicated: ^s^, *, **, and *** significant at 10%, 5%, 1%, and 0.1% level of significance, respectively. Complete statistical analysis results, including exact adjusted p values can be found in **Supplementary Table S6**. **(B)** Significant changes in the estimated absolute abundance of different bacterial families in the sand fly midgut early after *versus* before *Leishmania* infection (left panel), late after *versus* before *Leishmania* infection (center panel), and late *versus* early after *Leishmania* infection (right panel). Data are represented by effect size (LogFC) and Standard Error bars (two-sided; Bonferroni adjusted) derived from the ANCOM-BC model. All effect sizes with adjusted p < 0.1 (q value) are indicated: ^s^, *, **, and *** significant at 10%, 5%, 1%, and 0.1% level of significance, respectively. Complete statistical analysis results, including exact adjusted p values can be found in **Supplementary Table S7**. All results were obtained in three independent experiments.

Last, we focused on differences in estimated absolute abundance values (ANCOM-BC analysis) looking for potential markers of early and/or late infection. A few of the above-reported apparent relative abundance differences were still noted in the context of absolute numbers (**Figures 3A, B**; **Supplementary Tables S6**, **S7**). For instance, a significant decrease in the number of Microbacteriaceae late after versus before infection was noted (**Figure 3B**; **Supplementary Table S7**). More so, a significant increase in the number of *Ralstonia*, and consequently of Burkholderiaceae at the family level, as well as of *Sphingomonas* (only at the genus level in this case) were detected late versus early after infection (**Figures 3A, B**; **Supplementary Tables S6**, **S7**). Interestingly, via this estimated absolute abundance-based differential analysis, nine new genera/families were noted to change significantly in the context of *Leishmania* infection. *Cutibacterium* and *Porphyromonas* (Propionibacteriaceae and Porphyromonadaceae, respectively, at the family level) showed significantly lower numbers early after versus before infection (**Figures 3A, B**; **Supplementary Tables S6**, **S7**). *Corynebacterium* (and Corynebacteriaceae at the family level) also showed significantly lower numbers early after versus before infection, as well as late after versus before infection, making this genus/family a potential marker of non-infected sand flies (**Figures 3A, B**; **Supplementary Tables S6**, **S7**). Conversely, the genus *Enterococcus* (and the family Enterococcaceae) showed significantly higher absolute abundance both early and late after infection, highlighting them as potential general markers of *Leishmania*-infected sand flies in our experimental context (**Figures 3A, B**; **Supplementary Tables S6**, **S7**). More so, our results also revealed a decrease in the numbers of Peptostreptococcaceae late after versus before infection (**Figure 3B**; **Supplementary Table S7**). Importantly, we also detected significant changes when we compared the late versus early after infection statuses. In this context, we observed a significant increase in the absolute numbers of the *Streptococcus* and *Abiotrophia* genera (and the Streptococcaceae and Aerococcaceae families, respectively), as well as of the order Saccharimonadales, to which we could not assign a family/genera (**Figures 3A, B**; **Supplementary Tables S6**, **S7**). Overall, these results further support the notion that the gut microbiota of *P. duboscqi* sand flies changes dramatically in the context of *Leishmania* infection.

## 4 Discussion

With this study, we aimed to characterize the gut microbiota of *P. duboscqi* sand flies at the steady-state, and after *Leishmania* infection. Overall, we show that the composition of the gut microbiota of *P. duboscqi* sand flies changes significantly over the course of an infection with *L. major* parasites.

Curiously, we observed a decrease in the number of observed ASVs both early (D2; significantly), and late (D12; tendentiously) after infection. These results are in line with those previously reported for *Lu. longipalpis* sand flies infected with *L. infantum* parasites, both in the context of observed operational taxonomic units - OTUs - and of phylogenetic diversity (9). Together, both studies point to two distinct events of “microbial richness loss”, likely driven by selective pressures of different origins. The first decrease in richness is probably a result of the apport of new nutrients after the ingestion of blood by the sand fly. The second can be a consequence of bacteria-bacteria/bacteria-parasite competition for limited resources within a midgut now populated by high *Leishmania* numbers, and/or the result of a re-arrangement of microbial communities shaped by *Leishmania* excreted/secreted (by)products. Of note, our beta diversity results do not show a recovery in the composition of the sand fly gut microbiota after the defecation of blood meal remnants (before versus late after infection), further supporting the occurrence of independent selective pressure events that shape the midgut microbiota of adult *Leishmania*-infected female sand flies.

We also detected changes in the relative abundance of different bacterial genera/families. We can try to establish some parallels with the published bibliography. For instance, an apparent dominance of *Ochrobactrum* spp. in the gut microbiota of *P. duboscqi* sand flies was previously reported, including in blood-fed insects (17). A dominance of Rhizobiaceae (family *Ochrobactrum* spp. is part of), was also reported in the gut microbiota of *P. duboscqi* sand flies before, and after taking non-infected blood (15), as well as 14 days post-infection (10). Our data, showing the relative dominance of *Ochrobactrum* spp. early after infection, as well as a significant relative abundance both before and late after infection, may align with these published results. Of note, the first study only looked at culturable bacteria (17) and thus the relative values reported are probably over-estimated, while the second one was based on a limited number of samples (15), lacking the resolution necessary to account for the expected sample-to-sample heterogeneity. This said, other studies have reported the presence of *Ochrobactrum* spp. in sand fly larval rearing sites, and the ability of these bacteria to be transtadially transmitted from larvae to adults, explaining the presence of this bacterial genus in the gut microbiota of different lab-reared and wild-caught sand fly species (13, 35–38).

Additionally, the increase in the relative abundance of *Tsukamurella* spp. late after infection observed in this study was also previously reported in *Lu. longipalpis* sand flies infected with *L. infantum* parasites (9); the same parallel can be made with the Acetobacteraceae family. Of note, the *P. duboscqi* and *Lu. longipalpis* sand flies used in this study and in (9), respectively, are reared in the same environment and with the same food and sugar sources. However, while previously *Tsukamurella* spp. together with the Actinobacteria/Actinomycetota phylum (among others) were statistically defined, via linear discriminant analysis, as potential markers of infected sand flies (9), that was not the case in our context, after an ANCOM-BC analysis; for *Tsukamurella* spp. this was likely due to the observed sample heterogeneity. Instead, in this study, the genus *Enterococcus* and the family Enterococcaceae were defined as potential markers of infected sand flies, while the genus *Corynebacterium* and the family Corynebacteriaceae (curiously belonging to the Actinobacteria/Actinomycetota phylum) were defined as potential markers of non-infected sand flies. While we cannot exclude the hypothesis that these contradictory results may be just a consequence of the different statistical methods used in the two studies, overall, they seem to suggest that the diet is, likely, not the only factor that influences the microbiota of adult sand flies, in line with what was reported for mosquitoes (39). The hypothesis that, not only the genetic background, but also the infectious agent (different in the two contexts mentioned above) may condition the gut microbiota of adult sand flies is something to consider. A future side-by-side study of the gut microbiota of e.g *Lu. longipalpis* and *P. duboscqi* sand flies infected with *L. infantum* and *L. major* parasites, respectively (or even a more comprehensive study in the context of different sand fly species, infected with different *Leishmania* parasites, at the single-insect level) is warranted. Such study may address the above hypothesis, helping to disclose potential vector-parasite specific microbial signatures, but also, may simultaneously lead to the unveiling of potentially conserved microbiota signatures shared across different sand fly-*Leishmania* pairings.

Our ANCOM-BC analysis also revealed a significant higher absolute abundance of *Streptococcus*, *Sphingomonas*, *Ralstonia* and *Abiotrophia* spp. in sand flies late versus early after infection. The fact that these genera are more abundant in sand flies with heavier *Leishmania* infections may indicate a favorable parasite-bacteria relationship. These bacteria may proliferate in response to *Leishmania* growth, and/or they may be important to sustain the development of *Leishmania* parasites in the sand fly midgut. Of note, previous *Leishmania* infection studies in the context of antibiotic-treated sand flies, reported that *Leishmania* parasites need an undisturbed sand fly gut microbiota to establish themselves in the vector (9, 10). We can, therefore, speculate that some bacterial species within the abovementioned genera are *Leishmania* infection/metacyclogenesis promoters; none of these were previously identified as such. Future studies aiming at isolating these bacteria and characterizing their role in the context of *Leishmania* infection will help us to address this possibility. Of note, in this context there is a precedent. A study reported that *Serratia rubidaea* bacteria are *Leishmania* infection-enhancers in the context of sand fly gut dysbiosis (10). Notably, in our study *Serratia* spp. was the genus with the higher relative weight detected late after infection, and within the *Serratia* species we were able to attribute via our metagenomics analysis we found *Serratia rubidaea* (Data S1).

This study is not without limitations. For instance, our experimental settings differ from what is expected to occur in the field: i) sand flies take an infected bloodmeal from a living host (1) (different animals), and not via artificial membrane feeding; and ii) sand flies are expected to take multiple bloodmeals throughout their adult life span with consequences for vector competence (40). Future studies on the effect of multiple blood meals (different blood sources) on the gut microbiota of naturally infected sand flies are warranted. Additionally, we characterized the microbiota of the whole sand fly midgut, thus lacking the spatial resolution achieved elsewhere, where the microbiome of different midgut regions was analyzed separately (10). The use of higher depth techniques associated with a more compartmentalized analysis in a future study may provide useful insights into the dynamics of the sand fly gut microbiota as *Leishmania* spp. infection matures. Also, we analyzed the microbiota in the context of pooled samples and thus were neither able to look at potential individual variability, nor to establish potential associations between gut microbial composition and infection burden. To address this, we are now starting to study the gut microbiota of individual sand fly specimens, to undoubtedly establish bacteria-*Leishmania* interactions in the context of sand fly (mature) infections and potentially unveil new vector refractoriness-related intervention targets. Lastly, we only looked at the adult female sand fly and thus cannot know for sure the origin of the different gut microbiota components of our sand flies. More comprehensive studies, including not only the characterization of the gut microbiota of larval and pupal stages, but also of food, sugar, and blood sources (ideally under natural conditions, as reported elsewhere in the context of a different sand fly species (41), or trying to reproduce the sand fly-flora-habitat-reservoir interactions expected to happen in nature, but in the lab) are needed for the complete understanding of the life-long sand fly gut microbial dynamics.

All in all, our data contribute to the body of work in this field and may guide future studies aiming to: i) characterize different *Leishmania*-bacteria interactions in the sand fly midgut, ii) isolate bacteria beneficial and detrimental for the development of *Leishmania* parasites, and iii) leverage bacterial isolates/byproducts to manipulate the sand fly gut microbiota and negatively impact the development of *Leishmania* spp. parasites in their respective vectors.

## Data Availability

Publicly available datasets were analyzed in this study. These data can be found here: https://www.ncbi.nlm.nih.gov/bioproject/PRJNA1079352/con.

## References

[1] Cecilio P Cordeiro-da-Silva A Oliveira F . Sand flies: basic information on the vectors of leishmaniasis and their interactions with leishmania parasites. Commun Biol. (2022) 5:305. doi: 10.1038/s42003-022-03240-z, PMID: 35379881 PMC8979968

[2] Okwor I Uzonna J . Social and economic burden of human leishmaniasis. Am J Trop Med Hyg. (2016) 94:489–93. doi: 10.4269/ajtmh.15-0408, PMID: 26787156 PMC4775878

[3] Scheufele CJ Giesey RL Delost GR . The global, regional, and national burden of leishmaniasis: an ecologic analysis from the global burden of disease study 1990-2017. J Am Acad Dermatol. (2021) 84:1203–5. doi: 10.1016/j.jaad.2020.08.043, PMID: 32822799

[4] WHO . Who Leishmaniasis Fact Sheet (2024). Available online at: https://www.who.int/news-room/fact-sheets/detail/leishmaniasis (Accessed August 1, 2025).

[5] Cecílio P Oliveira F Cordeiro da Silva A . Vaccines for human leishmaniasis: where do we stand and what is still missing? In: Farhat A Hassan H , editors. Leishmaniases as Re-Emerging Diseases. IntechOpen, Rijeka (2018).

[6] Ponte-Sucre A Gamarro F Dujardin JC Barrett MP Lopez-Velez R Garcia-Hernandez R . Drug resistance and treatment failure in leishmaniasis: A 21st century challenge. PLoS Negl Trop Dis. (2017) 11:e0006052. doi: 10.1371/journal.pntd.0006052, PMID: 29240765 PMC5730103

[7] Balaska S Fotakis EA Chaskopoulou A Vontas J . Chemical control and insecticide resistance status of sand fly vectors worldwide. PLoS Negl Trop Dis. (2021) 15:e0009586. doi: 10.1371/journal.pntd.0009586, PMID: 34383751 PMC8360369

[8] Minwuyelet A Petronio GP Yewhalaw D Sciarretta A Magnifico I Nicolosi D . Symbiotic wolbachia in mosquitoes and its role in reducing the transmission of mosquito-borne diseases: updates and prospects. Front Microbiol. (2023) 14:1267832. doi: 10.3389/fmicb.2023.1267832, PMID: 37901801 PMC10612335

[9] Kelly PH Bahr SM Serafim TD Ajami NJ Petrosino JF Meneses C . The gut microbiome of the vector lutzomyia longipalpis is essential for survival of leishmania infantum. mBio. (2017) 8(1):e01121-16. doi: 10.1128/mBio.01121-16, PMID: 28096483 PMC5241394

[10] Louradour I Monteiro CC Inbar E Ghosh K Merkhofer R Lawyer P . The midgut microbiota plays an essential role in sand fly vector competence for leishmania major. Cell Microbiol. (2017) 19(10):10111. doi: 10.1111/cmi.12755, PMID: 28580630 PMC5587349

[11] Cecilio P Rogerio LA Serafim TD Tang K Willen L Iniguez E . Leishmania sand fly-transmission is disrupted by delftia tsuruhatensis tc1 bacteria. Nat Commun. (2025) 16(1):3571. doi: 10.1038/s41467-025-58769-4, PMID: 40341020 PMC12062286

[12] Vaselek S . Overview of microbial studies in sandflies and their progress toward development of paratransgenic approach for the control of leishmania sp. Front Trop Dis. (2024) 5:1369077. doi: 10.3389/fitd.2024.1369077

[13] Telleria EL Martins-da-Silva A Tempone AJ Traub-Cseko YM . Leishmania, microbiota and sand fly immunity. Parasitology. (2018) 145:1336–53. doi: 10.1017/S0031182018001014, PMID: 29921334 PMC6137379

[14] Cecilio P Oristian J Meneses C Serafim TD Valenzuela JG Cordeiro da Silva A . Engineering a vector-based pan-leishmania vaccine for humans: proof of principle. Sci Rep. (2020) 10:18653. doi: 10.1038/s41598-020-75410-0, PMID: 33122717 PMC7596519

[15] Tabbabi A Mizushima D Yamamoto DS Kato H . Effects of host species on microbiota composition in phlebotomus and lutzomyia sand flies. Parasit Vectors. (2023) 16:310. doi: 10.1186/s13071-023-05939-2, PMID: 37653518 PMC10472604

[16] Guernaoui S Garcia D Gazanion E Ouhdouch Y Boumezzough A Pesson B . Bacterial flora as indicated by pcr-temperature gradient gel electrophoresis (Tgge) of 16s rdna gene fragments from isolated guts of phlebotomine sand flies (Diptera: psychodidae). J Vector Ecol. (2011) 36 Suppl 1:S144–7. doi: 10.1111/j.1948-7134.2011.00124.x, PMID: 21366767

[17] Volf P Kiewegova A Nemec A . Bacterial colonisation in the gut of phlebotomus duboseqi (Diptera: psychodidae): transtadial passage and the role of female diet. Folia Parasitol (Praha). (2002) 49:73–7. doi: 10.14411/fp.2002.014, PMID: 11993554

[18] Cecilio P Pires A Valenzuela JG Pimenta PFP Cordeiro-da-Silva A Secundino NFC . Exploring lutzomyia longipalpis sand fly vector competence for leishmania major parasites. J Infect Dis. (2020) 222:1199–203. doi: 10.1093/infdis/jiaa203, PMID: 32328656 PMC7459136

[19] Lawyer P Killick-Kendrick M Rowland T Rowton E Volf P . Laboratory colonization and mass rearing of phlebotomine sand flies (Diptera, psychodidae). Parasite. (2017) 24:42. doi: 10.1051/parasite/2017041, PMID: 29139377 PMC5687099

[20] DeSouza-Vieira T Iniguez E Serafim TD de Castro W Karmakar S Disotuar MM . Heme oxygenase-1 induction by blood-feeding arthropods controls skin inflammation and promotes disease tolerance. Cell Rep. (2020) 33:108317. doi: 10.1016/j.celrep.2020.108317, PMID: 33113362

[21] Serafim TD Iniguez E Barletta ABF Cecilio P Doehl JSP Short M . Leishmania genetic exchange is mediated by igm natural antibodies. Nature. (2023) 623:149–56. doi: 10.1038/s41586-023-06655-8, PMID: 37880367

[22] Bolyen E Rideout JR Dillon MR Bokulich NA Abnet CC Al-Ghalith GA . Reproducible, interactive, scalable and extensible microbiome data science using qiime 2. Nat Biotechnol. (2019) 37:852–7. doi: 10.1038/s41587-019-0209-9, PMID: 31341288 PMC7015180

[23] Callahan BJ McMurdie PJ Rosen MJ Han AW Johnson AJ Holmes SP . Dada2: high-resolution sample inference from illumina amplicon data. Nat Methods. (2016) 13:581–3. doi: 10.1038/nmeth.3869, PMID: 27214047 PMC4927377

[24] Price MN Dehal PS Arkin AP . Fasttree 2–approximately maximum-likelihood trees for large alignments. PLoS One. (2010) 5:e9490. doi: 10.1371/journal.pone.0009490, PMID: 20224823 PMC2835736

[25] Katoh K Standley DM . Mafft multiple sequence alignment software version 7: improvements in performance and usability. Mol Biol Evol. (2013) 30:772–80. doi: 10.1093/molbev/mst010, PMID: 23329690 PMC3603318

[26] Yilmaz P Parfrey LW Yarza P Gerken J Pruesse E Quast C . The silva and “All-species living tree project (Ltp)” Taxonomic frameworks. Nucleic Acids Res. (2014) 42:D643–8. doi: 10.1093/nar/gkt1209, PMID: 24293649 PMC3965112

[27] Shannon CE Weaver W . The Mathematical Theory of Communication. Urbana: University of Illinois Press (1949). 117 p. p.

[28] Faith DP . Conservation evaluation and phylogenetic diversity. Biol Conserv. (1992) 61:1–10. doi: 10.1016/0006-3207(92)91201-3

[29] Pielou EC . The measurement of diversity in different types of biological collections. J Theor Biol. (1966) 13:131–44. doi: 10.1016/0022-5193(66)90013-0

[30] Chen J Bittinger K Charlson ES Hoffmann C Lewis J Wu GD . Associating microbiome composition with environmental covariates using generalized unifrac distances. Bioinformatics. (2012) 28:2106–13. doi: 10.1093/bioinformatics/bts342, PMID: 22711789 PMC3413390

[31] Cailliez F . The analytical solution of the additive constant problem. Psychometrika. (1983) 48:305–8. doi: 10.1007/BF02294026

[32] Anderson MJ . A new method for non-parametric multivariate analysis of variance. Austral Ecol. (2001) 26:32–46. doi: 10.1111/j.1442-9993.2001.01070.pp.x

[33] Anderson MJ Ellingsen KE McArdle BH . Multivariate dispersion as a measure of beta diversity. Ecol Lett. (2006) 9:683–93. doi: 10.1111/j.1461-0248.2006.00926.x, PMID: 16706913

[34] Lin H Peddada SD . Analysis of compositions of microbiomes with bias correction. Nat Commun. (2020) 11:3514. doi: 10.1038/s41467-020-17041-7, PMID: 32665548 PMC7360769

[35] Karakus M Karabey B Orcun Kalkan S Ozdemir G Oguz G Erisoz Kasap O . Midgut bacterial diversity of wild populations of phlebotomus (P.) papatasi, the vector of zoonotic cutaneous leishmaniasis (Zcl) in Turkey. Sci Rep. (2017) 7:14812. doi: 10.1038/s41598-017-13948-2, PMID: 29093481 PMC5665960

[36] Monteiro CC Villegas LE Campolina TB Pires AC Miranda JC Pimenta PF . Bacterial diversity of the american sand fly lutzomyia intermedia using high-throughput metagenomic sequencing. Parasit Vectors. (2016) 9:480. doi: 10.1186/s13071-016-1767-z, PMID: 27581188 PMC5007851

[37] Vaselek S Sarac BE Uzunkaya AD Yilmaz A Karaaslan C Alten B . Identification of ochrobactrum as a bacteria with transstadial transmission and potential for application in paratransgenic control of leishmaniasis. Parasitol Res. (2024) 123:82. doi: 10.1007/s00436-023-08087-9, PMID: 38175278

[38] Vivero RJ Castaneda-Monsalve VA Romero LR DH G Cadavid-Restrepo G Moreno-Herrera CX . Gut microbiota dynamics in natural populations of pintomyia evansi under experimental infection with leishmania infantum. Microorganisms. (2021) 9(6):1214. doi: 10.3390/microorganisms9061214, PMID: 34199688 PMC8228094

[39] Saab SA Dohna HZ Nilsson LKJ Onorati P Nakhleh J Terenius O . The environment and species affect gut bacteria composition in laboratory co-cultured anopheles Gambiae and aedes albopictus mosquitoes. Sci Rep. (2020) 10:3352. doi: 10.1038/s41598-020-60075-6, PMID: 32099004 PMC7042291

[40] Cecilio P Iniguez E Huffcutt P Ribeiro SP Kamhawi S Valenzuela JG . The impact of blood on vector-borne diseases with emphasis on mosquitoes and sand flies. Trends Parasitol. (2025) 41:196–209. doi: 10.1016/j.pt.2025.01.009, PMID: 39979193 PMC11998667

[41] Maleki-Ravasan N Oshaghi MA Afshar D Arandian MH Hajikhani S Akhavan AA . Aerobic bacterial flora of biotic and abiotic compartments of a hyperendemic zoonotic cutaneous leishmaniasis (Zcl) focus. Parasit Vectors. (2015) 8:63. doi: 10.1186/s13071-014-0517-3, PMID: 25630498 PMC4329651

