# Supplementary material for: Phlebotomus duboscqi gut microbiota dynamics in the context of Leishmania infection
Source: Front Immunol. 2026 Jan 5;16:1717935. doi: 10.3389/fimmu.2025.1717935 (PMC12812887; doi:10.3389/fimmu.2025.1717935)
Supplement: Supplementary file 2 [file Table1.docx]

Supplementary Materials for

*Phlebotomus duboscqi* gut microbiota dynamics in the context of *Leishmania* infection

Kristina Tang^1^, Yue Zhang^2#^, Claudio Meneses^1#^, Luana A. Rogerio^1^, Laura Willen^1^, Eva Iniguez^1^, Shaden Kamhawi^1^, Jesus G. Valenzuela^1^, Fabiano Oliveira^1*^, and Pedro Cecilio^3*^

^1^Vector Molecular Biology Section, Laboratory of Malaria and Vector Research, National; Institute of Allergy and Infectious Diseases, National Institutes of Health, Rockville, MD, USA;

^2^Integrated Data Sciences Section (IDSS), Research Technologies Branch, National Institute of Allergy and Infectious Diseases, National Institutes of Health, Rockville, MD, USA;

^3^Vector Biology Section, Laboratory of Malaria and Vector Research, National Institute of Allergy and Infectious Diseases, National Institutes of Health, Rockville, MD, USA;

^#^ These authors contributed equally to this work.

*** Correspondence:**Pedro Cecilio
[pedro.amadocecilio@nih.gov](mailto:pedro.amadocecilio@nih.gov)

and/or

Fabiano Oliveira

[loliveira@niaid.nih.gov](mailto:loliveira@niaid.nih.gov)

**This document includes:**

Supplementary Figures S1 and S2

Supplementary Tables S1 to S7

Data S1

**
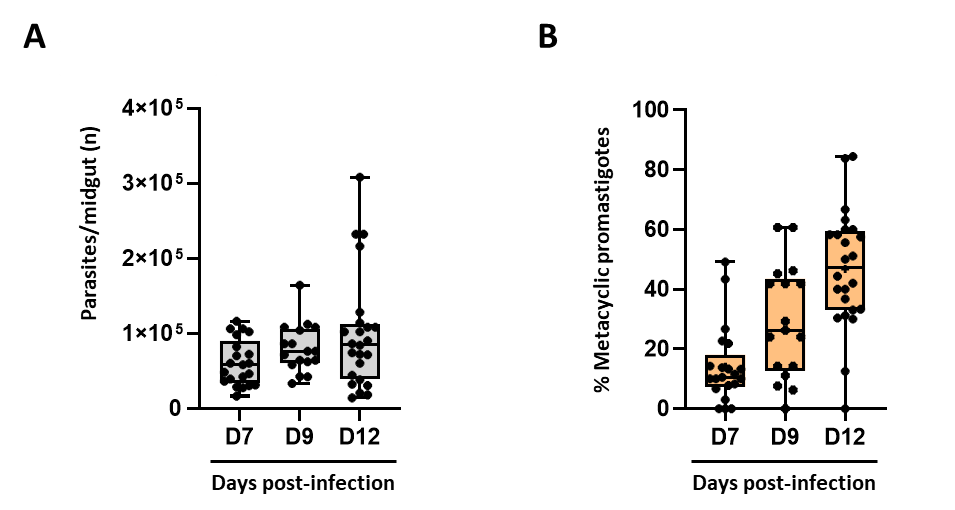
**

**Supplementary Figure S1. Quality control: sand fly infection status.** For the metagenomics analysis of *Leishmania*-infected sand flies, after an overnight starving period, *Phlebotomus duboscqi* sandflies were infected by artificial feeding with *Leishmania major* promastigotes. After infection, blood-fed females were sorted and kept on a 30% sucrose diet for up to 12 days. Midguts were dissected 7-, 9-, and 12-days post-bloodmeal and the infection burden was quantified microscopically for quality control purposes. (**A**) Total number of parasites per midgut of *L. major*-infected sand flies, 7-, 9-, and 11-days post-infection. (**B**) Percentage of metacyclic promastigotes per midgut of *L. major*-infected sand flies, 7-, 9-, and 11-days post-infection. Results are presented in the form of box and whiskers graphs (from maximum to minimum) overlapped with symbols referring to individual midgut data. All results were obtained in three independent experiments.

**
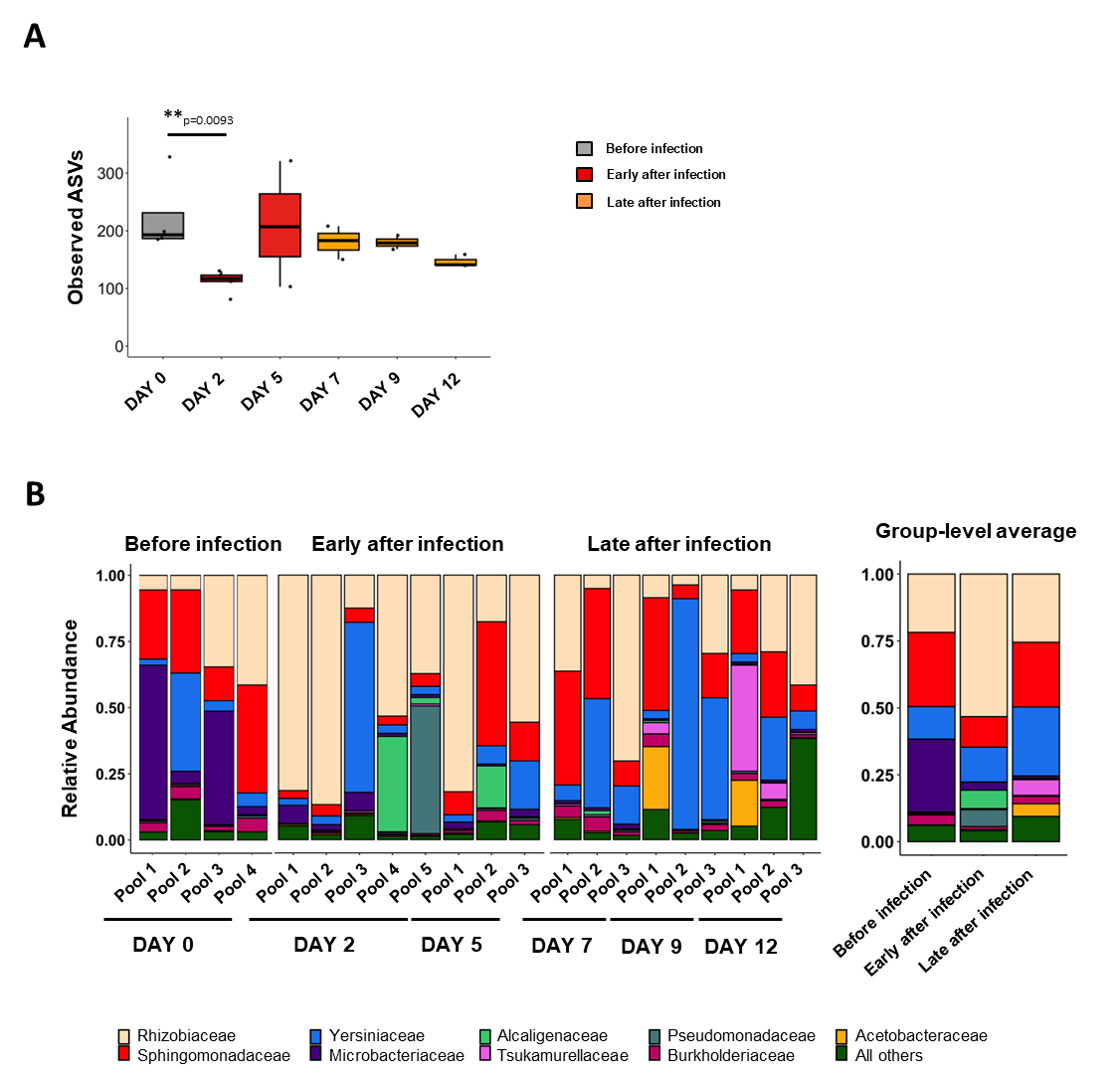
Supplementary Figure S2. The relative abundance of observed ASVs per time-point and relative abundance at the family level.** Pools of P. *duboscqi* sand fly midguts were collected one day before (day 0), as well as 2, 5, 7, 9, and 12 days after infection with *L. major* parasites and subjected to metagenomics analysis. In some instances, analysis was done in the frame of three major groups: before infection (day 0), early after infection (days 2 and 5), and late after infection (days 7, 9, and 12). (**A**) Variation in the number of observed Amplicon Sequence Variants ASVs in function of the collection time-point. Box-and-whisker graphs show an overview of the calculated values per pool of sand fly midguts collected before infection (grey; n=4), early after *Leishmania* infection (red; n=8), and late after *Leishmania* infection (orange; n=9). Statistical significance was determined using the Kruskal-Wallis test followed by post-hoc analysis and is highlighted. The complete statistical analysis results are listed in Table S2. (**B**) Relative abundance at the family level per sample pool and time-point (left panel), as well as per infection status (right panel). The most abundant genera are color-coded. All results were obtained in three independent experiments.

**Supplementary Table S1.** Observed ASVs at the group level: statistical analysis

| **Statistical analysis** | | | |  |
| --- | --- | --- | --- | --- |
| Kruskal-Wallis | | **p value** | **Significant?** |  |
|  |  | 0.044 | YES * |  |
| **Post-hoc analysis (multiple comparisons)** | | | |  |
| Dunn's test with p value adjustment by the holm method | | **p value** | **Significant?** |  |
| Group1 (n) | Group2 (n) |  |  |  |
|  |  |  |  |  |
| Before infection (4) | Early after infection (8) | 0.040838723 | YES * |  |
| Before infection (4) | Late after infection (9) | 0.30922063 | No |  |
| Early after infection (8) | Late after infection (9) | 0.30922063 | No |  |

**Supplementary Table S2.** Observed ASVs at the sample level: statistical analysis

| **Statistical analysis** | | | |  |
| --- | --- | --- | --- | --- |
| Kruskal-Wallis | | **p value** | **Significant?** |  |
|  |  | 0.035 | YES * |  |
| **Post-hoc analysis (multiple comparisons)** | | | |  |
| Dunn's test with p value adjustment by the holm method | | **p value** | **Significant?** |  |
| Group1 (n) | Group2 (n) |  |  |  |
|  |  |  |  |  |
| Day 0 (4) | Day 2 (5) | 0.009315256 | YES ** |  |
| Day 0 (4) | Day 5 (3) | 1 | No |  |
| Day 0 (4) | Day 7 (3) | 1 | No |  |
| Day 0 (4) | Day 9 (3) | 1 | No |  |
| Day 0 (4) | Day 12 (3) | 0.302909009 | No |  |

**Supplementary Table S3.** Relative abundance of different bacterial genera in the sand fly midgut in function of the infection status.

| **Genus** | **Average frequency** | | |
| --- | --- | --- | --- |
|  | **Before infection** | **Early after infection** | **Late after infection** |
| *Ochrobactrum* | 19.2727861 | 48.95067514 | 19.66886229 |
| *Sphingomonas* | 27.72359134 | 11.34435001 | 24.06344928 |
| *Serratia* | 11.98897221 | 13.05864478 | 25.77821661 |
| *Leifsonia* | 27.21022856 | 3.000370262 | 0.894335701 |
| *Tsukamurella* | 0.326397321 | 0.385522436 | 5.883565127 |
| *Pseudomonas* | 0.468164169 | 6.314712397 | 0.303103082 |
| *Ralstonia* | 3.647410837 | 1.110034424 | 2.770921081 |
| *Asaia* | 0.171454495 | 0.412624922 | 4.749875097 |
| Unknown | 3.415747128 | 11.37170267 | 6.56738169 |
| All others | 5.775247842 | 4.051362963 | 9.320290042 |
| Total | 100 | 100 | 100 |

|  | **Family** | **Average frequency (%)** | | |  |
| --- | --- | --- | --- | --- | --- |
|  |  | **Before infection** | **Early after infection** | **Late after infection** |  |
|  | Rhizobiaceae | 21.79873777 | 53.30667005 | 25.5372315 |  |
|  | Sphingomonadaceae | 27.7584493 | 11.37303695 | 24.13713098 |  |
|  | Yersiniaceae | 12.1345751 | 13.06464902 | 25.78244181 |  |
|  | Microbacteriaceae | 27.23057627 | 3.005623974 | 0.895373471 |  |
|  | Alcaligenaceae | 0.295375399 | 6.894955769 | 0.496499007 |  |
|  | Tsukamurellaceae | 0.326397321 | 0.385522436 | 5.883565127 |  |
|  | Pseudomonadaceae | 0.470332368 | 6.316463634 | 0.304214979 |  |
|  | Burkholderiaceae | 3.736140206 | 1.128213938 | 2.801238801 |  |
|  | Acetobacteraceae | 0.172788771 | 0.412624922 | 4.750319856 |  |
|  | All others | 6.076627483 | 4.112239316 | 9.411984469 |  |
|  | Total | 100 | 100 | 100 |  |
|  |  |  |  |  |  |
|  |  |  |  |  |  |

**Supplementary Table S4.** Relative abundance of different bacterial families in the sand fly midgut in function of the infection status.

**Supplementary Table S5.** Beta diversity: pairwise comparison analyses.

| **Statistical test** | **Pairwise Comparison** | **Adjusted p value*** |
| --- | --- | --- |
| PERMANOVA | Before infection Vs Early after infection | 0.024 |
|  | Before infection Vs Late after infection | 0.0959 |
|  | Early after infection Vs Late after infection | 0.054 |
| PERMDISP | Before infection Vs Early after infection | 0.2967 |
|  | Before infection Vs Late after infection | 0.2967 |
|  | Early after infection Vs Late after infection | 0.998 |
| **Note:** *p values adjusted using the Holm–Bonferroni method. | | |

**Supplementary Table S6.** Significant variation in bacterial absolute abundance values in the sand fly midgut in function of the infection status: ANCOM-BC analysis output at the Genus level, including statistics.

| **Comparison** | **Genus** | **LOG**  **(fold change)** | **Standard error** | **p value** | **q value** |
| --- | --- | --- | --- | --- | --- |
| Early after infection Vs Before infection | *Corynebacterium* | -2.550108675 | 0.486234759 | 1.56618E-07 | 2.42759E-05 |
|  | *Cutibacterium* | -1.253870294 | 0.35498539 | 0.000412157 | 0.062647939 |
|  | *Enterococcus* | 3.075213141 | 0.752029691 | 4.3283E-05 | 0.006622306 |
|  | *Porphyromonas* | -3.01254993 | 0.585223842 | 2.63716E-07 | 4.06123E-05 |
| Late after infection Vs Before infection | *Corynebacterium* | -1.977938971 | 0.534805636 | 0.000216941 | 0.039049331 |
|  | *Enterococcus* | 2.812635793 | 0.627916415 | 7.48827E-06 | 0.001355378 |
| Late after infection Vs Early after infection | *Abiotrophia* | 1.776974428 | 0.470850387 | 0.000160671 | 0.028920815 |
|  | *Ralstonia* | 1.166642739 | 0.271526217 | 1.73429E-05 | 0.003139057 |
|  | Saccharimonadales (Order) | 1.534455371 | 0.432579032 | 0.000389311 | 0.069297397 |
|  | *Sphingomonas* | 1.066516591 | 0.283731475 | 0.000170666 | 0.030549213 |
|  | *Streptococcus* | 1.7906524 | 0.336582165 | 1.03707E-07 | 1.88748E-05 |

**Supplementary Table S7.** Significant variation in bacterial absolute abundance values in the sand fly midgut in function of the infection status: ANCOM-BC analysis output at the Family level, including statistics.

| **Comparison** | **Family** | **LOG**  **(fold change)** | **Standard error** | **p value** | **q value** |
| --- | --- | --- | --- | --- | --- |
| Early after infection Vs Before infection | Corynebacteriaceae | -2.199585124 | 0.45028383 | 1.03489E-06 | 0.000101419 |
|  | Enterococcaceae | 3.075957675 | 0.805265376 | 0.000133557 | 0.012954987 |
|  | Porphyromonadaceae | -3.011805396 | 0.587245754 | 2.91755E-07 | 2.88837E-05 |
|  | Propionibacteriaceae | -1.248579804 | 0.371307405 | 0.000771959 | 0.074108108 |
| Late after infection Vs Before infection | Corynebacteriaceae | -1.763487427 | 0.426477328 | 3.54941E-05 | 0.003904355 |
|  | Enterococcaceae | 2.649873186 | 0.642371909 | 3.70511E-05 | 0.004038575 |
|  | Microbacteriaceae | -2.778810275 | 0.83056206 | 0.000820756 | 0.088641621 |
|  | Peptostreptococcaceae | -1.676604501 | 0.367609785 | 5.09526E-06 | 0.000565574 |
| Late after infection Vs Early after infection | Aerococcaceae | 1.670108796 | 0.473419972 | 0.000419103 | 0.046101309 |
|  | Burkholderiaceae | 1.058159138 | 0.280725702 | 0.00016366 | 0.018166249 |
|  | Streptococcaceae | 1.617334246 | 0.326269447 | 7.15712E-07 | 8.01598E-05 |

**Data S1. Assignment of ASVs, taxonomic details, and relative abundance per sample.** This table is available as a separate .xlsx file.
